# Early-Life Stress Affects Stress-Related Prefrontal Dopamine Activity in Healthy Adults, but Not in Individuals with Psychotic Disorder

**DOI:** 10.1371/journal.pone.0150746

**Published:** 2016-03-23

**Authors:** Zuzana Kasanova, Dennis Hernaus, Thomas Vaessen, Thérèse van Amelsvoort, Oliver Winz, Alexander Heinzel, Jens Pruessner, Felix M. Mottaghy, Dina Collip, Inez Myin-Germeys

**Affiliations:** 1 Department of Neuroscience, KU Leuven–University of Leuven, Leuven, Belgium; 2 Department of Psychiatry and Psychology, South Limburg Mental Health Research and Teaching Network, EURON, School for Mental Health and NeuroScience MHeNS Maastricht University, Maastricht, The Netherlands; 3 Department of Nuclear Medicine, Academic Medical Center, Amsterdam, The Netherlands; 4 Department of Nuclear Medicine, University Hospital RWTH Aachen University, Aachen, Germany; 5 Department of Psychiatry, Douglas Mental Health Institute, McGill University, Montreal, Quebec, Canada; 6 Department of Nuclear Medicine, Maastricht University Hospital, Maastricht, The Netherlands; Max Planck Institute of Psychiatry, GERMANY

## Abstract

Early life stress may have a lasting impact on the developmental programming of the dopamine (DA) system implicated in psychosis. Early adversity could promote resilience by calibrating the prefrontal stress-regulatory dopaminergic neurotransmission to improve the individual’s fit with the predicted stressful environment. Aberrant reactivity to such match between proximal and distal environments may, however, enhance psychosis disease risk. We explored the combined effects of childhood adversity and adult stress by exposing 12 unmedicated individuals with a diagnosis of non-affective psychotic disorder (NAPD) and 12 healthy controls (HC) to psychosocial stress during an [^18^F]fallypride positron emission tomography. Childhood trauma divided into early (ages 0–11 years) and late (12–18 years) was assessed retrospectively using a questionnaire. A significant group x childhood trauma interaction on the spatial extent of stress-related [^18^F]fallypride displacement was observed in the mPFC for early (b = -8.45, t(1,23) = -3.35, p = .004) and late childhood trauma (b = -7.86, t(1,23) = -2.48, p = .023). In healthy individuals, the spatial extent of mPFC DA activity under acute psychosocial stress was positively associated with the severity of early (b = 7.23, t(11) = 3.06, p = .016) as well as late childhood trauma (b = -7.86, t(1,23) = -2.48, p = .023). Additionally, a trend-level main effect of early childhood trauma on subjective stress response emerged within this group (b = -.7, t(11) = -2, p = .07), where higher early trauma correlated with lower subjective stress response to the task. In the NAPD group, childhood trauma was not associated with the spatial extent of the tracer displacement in mPFC (b = -1.22, t(11) = -0.67), nor was there a main effect of trauma on the subjective perception of stress within this group (b = .004, t(11) = .01, p = .99). These findings reveal a potential mechanism of neuroadaptation of prefrontal DA transmission to early life stress and suggest its role in resilience and vulnerability to psychosis.

## Introduction

Adverse early-life experiences such as abuse or parental loss are highly prevalent phenomena in children with reports of up to 60% being exposed to at least one major traumatic event by the time they are 16 years old [1]. These statistics become all the more concerning in the light of epidemiological evidence linking traumatic experiences in early life to higher risk for psychosis years later [2, 3]. Indeed, a comprehensive meta-analysis of case-control and population-based studies revealed a threefold increase in risk of developing a psychotic disorder among those reporting childhood trauma [4]. Moreover, compelling prospective evidence suggests a dose-response relationship between the exposure to early life trauma and incidence of psychotic symptoms [5] and the subsequent need for care [6]. Most individuals facing early adversity, however, are resilient to psychosis, and only a small portion descends into psychotic illness [7]^.^ Thus, studying the effects of childhood trauma in healthy adults and patients with psychotic disorder can potentially allow to identify some of the neurodevelopmental programming mechanisms fostering resilience to adversity.

Various lines of evidence suggest that psychosis is associated with critical alterations in central stress-regulatory mechanisms affecting neural and endocrine stress systems [8–10], and manifested through maladaptive affective and psychotic reactivity to stress [11, 12]. Resilience to psychosis, on the other hand, appears to be promoted by advantageous neuroadaptive changes in the stress-modulatory network, through which early life stress likely exerts a hormetic effect on stress susceptibility later in life [13]. The dopamine (DA) system, which has long been the subject of investigation in psychosis [14], has been implicated in these changes [15, 16], making it a prime candidate for explorations into the putative protective versus psychotogenic effects of early life stress.

Preclinical work has revealed the critical DAergic hubs of the stress network: the medial prefrontal cortex (mPFC), nucleus accumbens and striatum [15, 17–19], with recent work proposing a reciprocal relationship between these hubs [20, 21]. Several reports suggest that exposure to early life trauma may affect this pathway; rodents exposed to early adversity demonstrate long-lasting stress blunted mPFC DA outflow [22, 23] and increased tonic DA levels in subcortical areas [24]. Corroborating evidence in humans has also implied the DA system in stress processing in most of the hubs within the stress network, primarily mediated by D_2/3_ receptors [25–27]. Moreover, increased striatal DAergic reactivity to stress has been associated with both childhood adversity [28, 29] and the psychosis continuum [27, 29].

While there is rising support for the role of midbrain DA release in the psychotogenic effects of stress [27, 29], the evidence for the role of the mPFC remains inconclusive. Work of our group recently offered evidence for unaltered stress-related prefrontal DA function in psychosis: the DAergic response to psychological stress in mPFC was similar in healthy controls and patients with a psychotic disorder, and correlated with subjective experience of stress in the entire sample [25]. The effect of early life stress, however, was not taken into account, leaving possible resilience or vulnerbaility mechanisms unidentified.

The current study, therefore, aimed to investigate the effect of childhood adversity on DAergic stress processing in frontal cortical areas in non-medicated patients with non-affective psychotic disorder (NAPD) as well as in healthy volunteers (HV) in order to further elucidate the DAergic contribution to both vulnerability as well as resilience to psychosis. To this end, we used data acquired previously (25) in a single bolus-infusion [^18^F]fallypride positron emission tomography (PET) during which psychosocial stress was induced using a well-validated Montreal Imaging Stress Task (MIST; [28]). Conform previous reports implicating the mPFC in the traumagenic dysfunction and stress modulation [25, 30] the mPFC as a whole, as well as its ventral and dorsal portions, were a priori selected as the regions of interest (ROI). In these regions, we hypothesized a differential effect of childhood trauma on the spatial extent of stress-induced DA release among NAPD and HV. Moreover, differential effects of childhood trauma on the subjective experience of stress during the scan were expected in the two groups.

## Materials and Methods

### Sample characteristics

The sample consisted of 12 HV and 12 unmedicated NAPD matched on age, gender and education described in detail previously [25]. All NAPD were currently off antipsychotic medication (AP) for longer than one year, did not currently use mood stabilizers, antidepressants or benzodiazepines. Participants were recruited through regional and national media and, additionally, NAPD were recruited through local mental health services. Inclusion criteria independent of group: I) age 18–60 II) able to provide informed consent. Exclusion criteria independent of group: I) current/past use of illicit drugs according to the Composite International Diagnostic Interview (CIDI; WHO, 1990) (lifetime: >15 times cannabis, >5 times other drugs; illicit drug use in the past year), II) ferromagnetic metal element in or on the body, III) neurological disease, IV) pregnancy. HV-specific exclusion criteria: lifetime history of psychiatric illness according to the diagnostic and statistical manual of mental disorders (DSM) IV criteria and lifetime AP use. NAPD-specific inclusion criterion: diagnosis of non-affective psychosis according to DSM-IV criteria (NAPD were not in remission according to the Positive and Negative Syndrome Scale (PANSS) criteria [31]. On the day of scanning, a urine screening was performed to ascertain current drug use and pregnancy. The RWTH Aachen University ethics committee approved the study. PET approval was additionally granted by the national authority for radiation protection in humans in Germany (Bundesamt für Strahlenschutz, BfS). Written informed consent was obtained before participation, and participants were treated in accordance with the Declaration of Helsinki.

### Psychosocial stress paradigm

**Fig 1:**
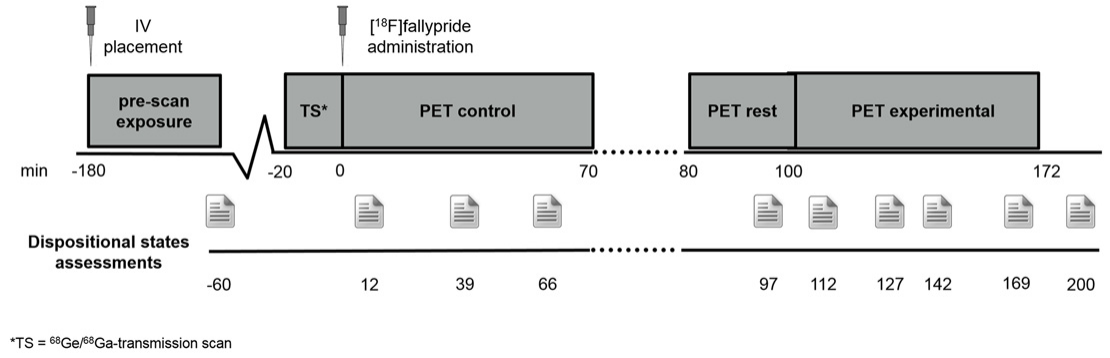
Schematic representation of the single bolus design.

Psychosocial stress was induced using the MIST [28], a mental arithmetic task with social evaluative component. During the MIST task, participants were asked to solve arithmetic problems first under a control condition during which no time constraint or feedback were present, and subsequently under the experimental condition where time and difficulty were automatically adjusted to ensure 30–40% error rate. Participants were continuously made aware of their suboptimal performance via a visual performance bar and scripted verbal negative feedback delivered approximately every 12 minutes throughout the experimental condition (6 times in total), during which a confederate researcher reminded the participants that they were performing worse than all previous participants. There were 10 6-minute blocks of MIST control and experimental version (Fig 1). Dispositional subjective stress and positive symptoms of psychosis were assessed pre scan (n = 1), during each PET condition (n = 8) and post scan (n = 1) (Fig 1) using validated 7-point Likert Scale items. Similar to previous work [11, 25, 30], subjective stress was measured using items with sufficient variability and internal consistency (Cronbach’s alpha = .69): “I feel pressured”, “I feel judged”,and “I’m in control” (recoded). Moreover, positive symptoms of psychosis were assessed using the items: “I hear voices”, “I see things” and “I feel suspicious” (Cronbach’s α = .7). Factor analyses confirmed that the subjective stress items loaded on a single factor, which was also the case for the psychotic symptoms items. Since the psychosocial stress task was always administered last, it might have been more demanding for patients than for controls, thus influencing their subjective state and brain activity. The perceived difficulty of the current task at hand was thus assessed with the item “this is difficult for me”, rated on the same scale.

### Image acquisition and analysys

#### MRI

T1-weighted Magnetic Resonance Imaging (MRI) scans were acquired on a 1.5T Philips (Philips Medical Systems. Herrsching, Germany) machine with TE = 4.59ms, TR = 30ms, matrix dimensions = 256x256, slice thickness = 2mm, slice number = 176. This scanner was replaced by a Siemens 3T scanner (Siemens Healthcare. Munich, Germany) and all remaining scans (39%) were collected using the Magnetization Prepared Rapid Acquisition Gradient-Echo (MP-RAGE) sequence, with TE = 2.52ms, TR = 1900ms, matrix dimensions = 256x256, slice thickness = 1mm, slice number = 176. A similar proportion of scans for HV and NAPD was collected on the second machine (5/12 vs. 4/12).

#### Tracer preparation

The radiosynthesis of [^18^F]fallypride was a high-yield modification of the synthesis method for [^18^F]desmethoxyfallypride, described in detail previously [32].

#### PET acquisition

PET measurements were performed in three-dimensional mode on a Siemens ECAT EXACT HR+ scanner (Siemens-CTY, Knoxville, TN, USA). Sixty-three slices of 2.425mm slice thickness (pixel size = 2mm x 2mm) were reconstructed per time frame by filtered back projection (Hamm filter) after Fourier rebinning into two-dimensional sinograms. Data sets were corrected for random coincidences, scatter radiation and attenuation (10min ^68^Ge/^68^Ga-transmission scan). Image matrix was 128x128. PET data were smoothed (4mm FWHM), realigned (realignment image based on first 15 minutes of the scan), co-registered (transformation matrix based on first 10 realigned frames to individual T1 MRI (PMOD v3.1, PMOD Technologies Ltd., Zurich, Switzerland) and normalized (SPM8, Wellcome Trust, UK). Preprocessing details have been published previousled [25]. Data were collected in two segments, a control and experimental part, in a single session with single bolus administration (Fig 1; [25, 33]).

#### PET analysis

Time-activity curves (TAC) were obtained for the cerebellum (reference region) and temporal and frontal regions. Mask preparation details have been described previously. Briefly, regions were based on Brodmann definitions. Masks were custom-tailored to the individual’s MRI, transferred to co-registered PET data in PMOD v3.1 and visually inspected for fit by two independent individuals. PET data were analyzed using a modified simplified reference tissue model (SRTM), in accordance with previous work [33–38]. Stress-induced [^18^F]fallypride displacement was quantified using TAC plots and receptor kinetic parameters. Tracer displacement was calculated for every person on a voxel-wise basis as the standardized value of γ (γ/std(γ); [38]), where γ is considered an additional time-varying parameter in the SRTM estimating the amplitude of ligand displacement at start of the experimental condition in a single scan session (based on the assumption that changes in competition between DA release and radioligand competition are reflected in the estimation of γ; [34]). γ was calculated over an exponential decay function h(t) = exp[−τ(t−T), where t = measurement time, T = time of experimental condition initiation and τ controls the rate at which activation effects die away (dissipation rate set to τ = 0.03 min^−1^; [33, 36, 37]). The number of voxels surviving p(/number of total voxels) = .05 reflects the spatial extent of task-induced ligand displacement and was used as primary outcome measure of stress-related DA function. This approach has been validated for [^18^F]fallypride [36] and has been used to investigate phasic DAergic activity in extrastriatal areas [33, 38].

### Childhood trauma assessment

Childhood trauma was measured using Childhood Experience of Care and Abuse (CECA-Q; [39]), a validated, retrospective questionnaire to assess childhood trauma in early childhood spanning from 0 to 11 years of age, and late childhood encompassing years 12 through 17. For the purpose of this study, a composite score was created for each time period using 15 dichotomous (‘yes’ = 1 and ‘no’ = 0) items informing about family arrangements, parental loss, physical and sexual abuse, neglect and bullying.

### Analyses

Final analyses were performed using STATA 11.2 (StataCorp, 2011). The percentage of voxels in a ROI surviving the Bonferroni-corrected threshold was used as outcome for stress-induced changes in DAergic activity [30, 36, 38]. Conform previous results from this sample [25], the mPFC was a priori selected as the primary ROI, with its ventral and dorsal portions (vmPFC and dmPFC, respectively) as two additional ROIs. The percentage of voxels of the left and right regions were summed into a corresponding bilateral ROI and entered into regression analyses as the dependent variable, with the childhood trauma score as the predictor and group (NAPD, HV) as the interaction term. Separate linear regressions were performed for each ROI, and for each trauma timeframe: early and late. Assessments of subjective stress and psychotic symptoms during the scan were averaged for the control and experimental part, and the difference score (experimental-control) was used as the outcome variable in regression analyses, with childhood trauma scores entered as predictors. To compare the two groups on the change in perceived difficulty of the task, this variable was averaged for the control and experimental part, the difference score was computed as above, and entered in a regression analysis as the outcome, with group (NAPD, HV) as the predictor. Regression analyses were corrected for age and gender.

## Results

### Sample demographics

As decribed in detail previously [25], HV and NAPD were matched on age (M = 48.08 (SD = 9.94) vs. M = 44.67 (SD = 11.24)), gender (8 male, 4 female per group), education, lifetime drug use and smoking frequency (all n.s.). Four NAPD were antipsychotics (AP)–naïve; the remainder of the sample were off AP for 7.09 (SD = 4.96) years. Patients endorsed moderate levels of positive symptoms of psychosis (PANSS positive symptom scale mean = 11.83, SD = 3.93). The ability of the MIST to successfully induce stress and temporarily increase positive psychotic symptoms in this sample has been reported previously [25]. Additionally, the ratings of the perceived difficulty of the task increased numerically from control to experimental condition for both HV (M = 1.63, SD = 1.76) and NAPD (M = 1.54, SD = 1.40) to the same extent (b = -.17, t(1,23) = -.25, p = .807). Healthy participants endorsed a mean of 2.42 (SD = 1.51) adverse events in early childhood and 2.67 (SD = 1.5) adverse events in late childhood. NAPD scored 3.42 (SD = 1.88) and 2.75 (SD = 1.36) for early and late childhood trauma respectively. The two groups did not differ in early (t(1,23) = -1.31, p = .20) nor late childhood trauma scores (t(1,23) = -.29, p = .77).

### The effect of childhood trauma on stress-induced [^18^F]fallypride displacement

**Fig 2:**
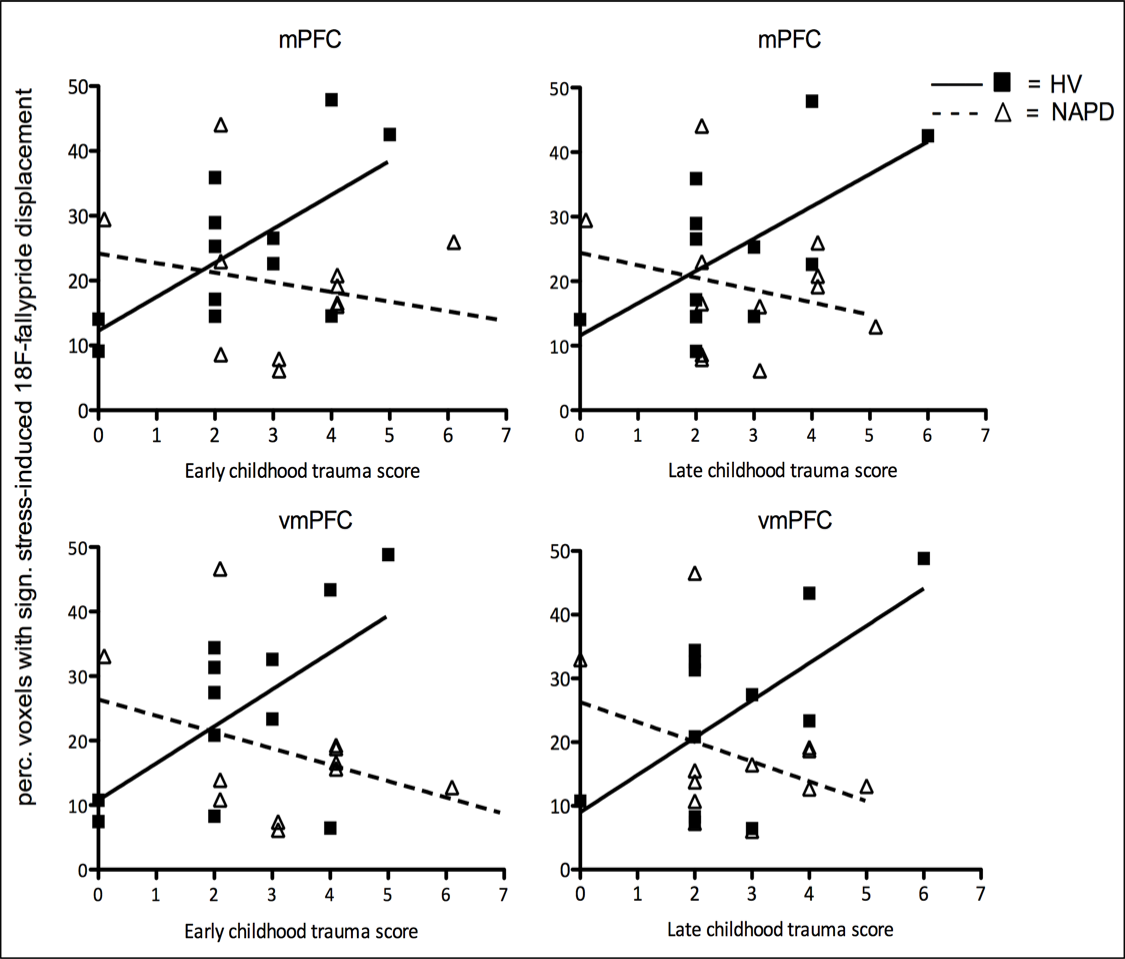
The effect of childhood trauma on spatial extent of DA activity in mPFC and vmPFC. Early (ages 0–11) and late (ages 12–17) childhood trauma scores (x-axis) were associated with increased spatial extent of stress-induced mPFC and vmPFC [^18^F]fallypride displacement (y-axis) in HV. No such associations were observed in NAPD. Association in HV significant at p<0.05.

**Fig 3:**
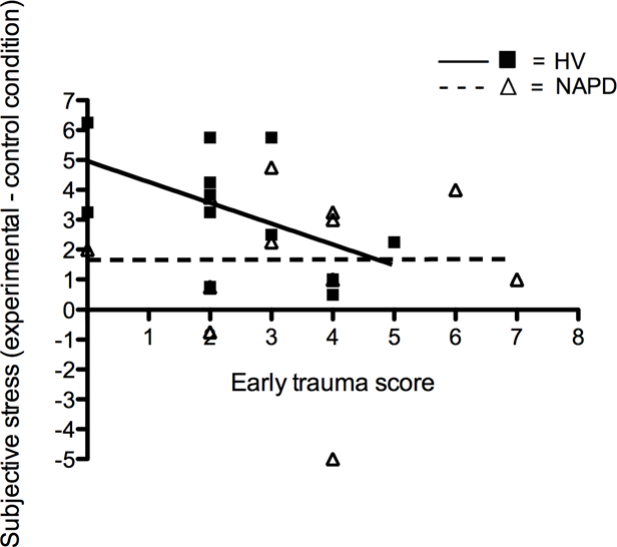
Correlation between early childhood trauma scores and subjective stress during PET. A trend-level association between early childhood trauma scores (0–11) and decreased reactivity to the stress task in HV, but not NAPD. Association in HV p = 0.07.

Binding potential relative to non-displaceable binding potential (BP_ND_) calculated over the complete paradigm using the SRTM [40] in mPFC was .51 (SD = .2). As evidenced by Fig 2, a significant group x childhood trauma interaction on the spatial extent of stress-related [^18^F]fallypride displacement was observed in the mPFC for early childhood trauma (b = -8.45, t(1,23) = -3.35, p = .004), and late childhood trauma (b = -7.86, t(1,23) = -2.48, p = .023). Within the control group, a significant positive association emerged between the spatial extent of stress-induced tracer displacement in the mPFC and early childhood trauma (b = 7.23, t(11) = 3.06, p = .016; Figs 2 and 3) and late childhood trauma scores (b = 5.47, t(11) = 2.54, p = .035; Fig 2). In the patient group, there was no association between childhood trauma and the spatial extent of the tracer displacement in mPFC (early b = -1.22, t(11) = -0.67, p = .519; late b = -1.68, t(11) = -.68, p = .513; Figs 2 and 3).

An analogous significant group x childhood trauma interaction effect was observed in the vmPFC (early b = -9.53, t(1,23) = -3.15, p = .006; late b = -9.5, t(1,23) = -2.61, p = .018; Fig 2). In healthy controls, a trend-level positive association between the spatial extent of stress-induced tracer displacement in the vmPFC and childhood trauma was observed for both early trauma (b = 7.2, t(11) = 2.22, p = .058) and late trauma scores (b = 6.02, t(11) = 2.24, p = .056). Similarly to the mPFC, there was no significant association between tracer displacement in the vmPFC and childhood trauma in the patient group (early b = -2.18, t(11) = -1.15, p = .282; late b = -2.7, t(11) = -1.03, p = .331; Fig 2). There was no main effect, interaction effect or within-group associations between the two childhood trauma scores and stress-related fallypride displacement in the dmPFC (all p-values>.05).

### The effect of childhood trauma on subjective stress and psychopathology

The association between early trauma score and subjective stress scores during the MIST paradigm was not significantly different for NAPD and HV (b = .7, t(1, 23) = 1.18, p = .26). However, when testing within-group main effect of childhood trauma on subjective experience of stress, a trend for a negative association between early childhood trauma and the subjective stress response was detected in HV (b = -.7, t(11) = -2, p = .07), with higher early childhood trauma being associated with lower subjective stress responses to the task (Fig 3). No main effect of childhood trauma on the subjective stress response to the task was present in NAPD (b = .004, t(11) = .01, p = .99; Fig 3). There was no interaction or main effect between late childhood trauma and the subjective stress response to the task (all p-values>.05) and there was no association between the two childhood trauma scores and psychotic symptoms during the scan in NAPD.

## Discussion

We examined the association between childhood trauma and stress-induced prefrontal DA activity of healthy individuals and patients with psychotic disorder using [^18^F]fallypride PET. We observed a significant difference in the association between childhood trauma score and spatial extent of stress-induced prefrontal DA activity in each group; In healthy subjects, severity of childhood trauma was associated with more extensive stress-related DA activity in mPFC. This effect was especially pronounced in relation to early childhood trauma, and largely driven by DA activity in the ventral portion of mPFC. Contrarily, in the patient group, there was no association between childhood trauma and the spatial extent of stress-related DA activity in this region, and this was the case for its ventral and dorsal portions, as well as for early and late childhood trauma. While the interaction between group and childhood trauma on behavioral stress response was not significant, a trend for a main effect of early trauma emerged in the control group, where increased exposure to early trauma was associated with decreased subjective stress responses to the task. No main effect of childhood trauma on subjective experience of stress was detected in individuals with psychotic disorder.

### Healthy individuals

These results first of all implicate prefrontal DA transmission in the human stress response and confirm the role of the mPFC in this function. Furthermore, they build upon our previous findings of increased DA activity in mPFC under acute psychosocial stress in this [25] and another sample [30], by showing that in the healthy brain, distal forms of stress impact the acute prefrontal DAergic stress response. The results presented in this manuscript suggest that increased DAergic activity observed in the striatum of those exposed to childhood adversity [28] also extends to the cortex.

The positive association between childhood trauma and the spatial extent of mPFC DA activity under stress in healthy adults could be interpreted as one of the mechanisms of adaptive neuroplasticity in the mPFC [16], characterizing resilience. The within-group behavioral results suggest that this mechanism may underlie increased robustness to psychopathology, as more severe trauma was associated with decreased sensitvity to the experimental stressor.

This notion corroborates the emerging evolutionary perspective on resilience to psychopathology which maintains that early life adversity could induce adaptive changes that optimize the individual’s fit with the predicted (adverse) environment [13]. That is, stress during development could “inoculate” certain individuals to better cope with challenges encountered during adulthood [7], such as those evoked by the present experiment.

While social support, parenting and other external circumstances undoubtedly play a role, genetic makeup is thought to largely determine a stress-vulnerable versus stress-resilient phenotype [8]. Neuroadaptation to stress is multifold, and believed to involve variation in the glucocorticoid receptor (GR; [41] and catechol-O-methyltransferase (COMT) expression, both of which directly influence prefrontal DA function [42]. Stress has been shown to exclusively activate the GRs located on mPFC DA neurons leading to DA efflux, which in turn mediates DA release downstream [43]. In the interaction with childhood trauma, common variants of the GR gene predict increased biomarkers for, and actual vulnerability to, psychopathology in adulthood [44]. Meanwhile, COMT genotype predicts the extent of prefrontal DA activity and stress-sensitivity [38], and is reported to modulate the effect of childhood trauma on cognition and symptoms of psychosis [45]. Collectively, these studies offer one possible explanation of how childhood adversity in interaction with (epi)genetically optimized prefrontal DA reactivity to stress may confer a resilient phenotype.

### Individuals with psychotic disorder

In NAPD, on the other hand, there was no association between childhood trauma and the spatial extent of stress-related DAergic activity in this region, and this was the case for its ventral and dorsal portions, as well as for early and late childhood trauma. Moreover, no within-group association between childhood trauma and subjective stress response to the task was observed. Seeing that this pattern deviates from the putative adaptive DA response of HV, one could reasonably speculate that in individuals that develop psychosis later lin life, childhood trauma fails to evoke the necessary calibration of the DA system to better endure stress [8]. From the large-scale brain network perspective, the dysregulation of the prefrontal node by childhood trauma could have a noxious effect on stress responsiveness in the interconnected subcortical hubs [46]. This notion is supported by reports from other groups implicating the striatum in aberrant reactivity to stress in psychosis in general [27] and in the pathogenesis of psychosis in particular [47, 48]. Furthermore, low maternal care in the early life has been shown to be more prevalent in individuals with schizotypy and associated with increased stress-induced DA release in the striatum [29].

The underlying mechanism of such divergent trajectories of individuals with psychosis exposed to similar levels of childhood trauma as their healthy counterparts could be attributable to a stress-susceptible genetic make-up. In addition to studies implicating GR and COMT variants in poor outcomes following childhood trauma discussed earlier, an extensive general population study identified the COMT polymorphism as significant moderator of the susceptibility to psychotic experiences following childhood maltreatment [49]. Meanwhile, patients with psychosis carrying the COMT Met/Met genotype demonstrated increased affective and psychotic reactivity to stress [50]. Collectively, these studies support the existence of a stress-vulnerable genotype implicating prefrontal DA function and warrant integrated exploration of the trauma-stress-psychosis connection.

### Strengths and limitations

A number of strengths and limitations regarding the design and methodology of the current study were previously discussed in detail elsewhere[25].

The most important consideration includes the fixed order of the control-experimental condition to accommodate the model and prevent the long-lasting effects of stress from contaminating the control condition. It is possible that this design could introduce an order effect due to, for instance, greater proneness to fatigue in the patient group. Although a recent [^18^F]fallypride PET experiment that employed the MIST reported a main effect of stress on mPFC DA activity irrespective of the condition order [26], the two conditions were administered on separate days, and the population under study only included healthy controls. While in the current study patients did not endorse greater increase in perceived difficulty of the stress than the controls did, it still does not rule out the possibility that they were more fatigued by it or more reactive to it in some other way.

Other limitations specific to this article include the variation in BD present in both groups that suggests that although the DA system plays an important role, other factors likely also contribute to resilience and vulnerability to stress. Additionally, the relatively small sample size could both preclude and inflate the subtle effect of childhood trauma on the arguably noisy DA-ergic neurotransmission of the 12 patients included in this study. According to our post-hoc power calculation, doubling this sample size would yield moderate-to-high power in future exploration of this intriguing phenomenon.

Another limitation pertains to the CECA questionnaire used to quantify childhood trauma. The retrospective nature of the self-report of stressful childhood experiences is subject to recall bias. However, this questionnaire has been well-validated and widely-accepted as an accurate and reliable index of exposure to adversity in childhood [51, 52]. Moreover, the structure of the CECA questionnaire used in the current study only allows for quantification of the stressful life events, but does not allow to qualify the frequency and gravity of the event self. This compromise has been introduced in order to minimize memory bias, as it has been shown that the recollection of an event is more susceptible to forgetting and false memory formation than a mere recognition of a presence or absence of the event [53, 54].
